# Medical error in treatment of *Amanita phalloides* poisoning in pre-hospital care

**DOI:** 10.1186/s13049-022-01008-2

**Published:** 2022-03-19

**Authors:** Anna Smędra, Katarzyna Wochna, Dariusz Zawadzki, Jarosław Berent

**Affiliations:** 1 Chair and Department of Forensic Medicine, Medical University of Lodz, ul. Sędziowska 18a, 91-304 Lodz, Poland; 2 Department of Emergency Medicine for Children, II Chair of Paediatrics, Medical University of Lodz, Lodz, Poland

**Keywords:** Mushroom poisoning, *Amanita phalloides*, Pre-hospital care, Correctness of medical treatment, Medical error, Refugees

## Abstract

**Background:**

Geopolitical and climate changes form the background of the current migration crisis. It has many faces. One of them are the tragic cases of poisoning of refugees due to eating wild forest mushrooms for socioeconomic reasons in the Western and Northern European countries. The most serious food poisonings in Europe, but not only, are caused by lamellar mushrooms, the most dangerous being *Amanita phalloides*. Its poisonous properties can be attributed to α-amanitin, an RNA polymerase II inhibitor. Unfortunately, as it is characterized by a delayed onset of symptoms, *A. phalloides* poisoning has a high risk of complications.

**Case presentation:**

Our article presents a case of *A. phalloides* poisoning in a 28-year-old man, in which the responding medical emergency unit made errors in diagnosis and treatment. Since the correct diagnosis was made too late, the typical treatment of *A. phalloides* poisoning was ineffective. The patient suffered a life-threatening liver failure and needed liver transplant from a deceased donor.

**Conclusions:**

Mushroom poisoning is a particularly important problem not only in countries with a mushroom picking tradition, but also—due to the inflow of refugees—in countries where mushroom poisoning was very rare until recently. In such cases it is crucial to quickly implement the correct procedure, as this can prevent the need for liver transplant or even death. This is a particularly important consideration for the first medical professionals to contact the patient, especially in cases where the patient reports mushrooms consumption and presents alarming symptoms of the gastrointestinal tract. Such situations cannot be underestimated and ignored.

## Introduction

### Background

The kingdom Fungi constitutes a huge and overlooked source of agents with extensive health benefits. There is a vast body of evidence indicating that mushrooms demonstrate immunomodulatory, antiviral, antibacterial, antidiabetic, antitumor, antioxidant, and hypocholesterolemic effects [1–5]. But aside from the positive effects, they can also be toxic.

Geopolitical and climate changes that form the background of the current migration crisis [6] unexpectedly also caused the problem of mushroom poisoning in countries where it was not a major problem so far. Due to low socioeconomic level and hunger refugees collect and eat mushrooms, and thus the mushroom poisoning phenomenon is observed more and more frequently [7]. In 2016 Azerbaijan Medical Association Journal published a paper on the death of 6 Syrian asylum seekers after eating poisonous mushrooms specified as *Amanita phalloides* [8]. Also, a Danish media report from 2017 pertained to the death of two children and hospitalization of nine other family members of the same family, possibly from Congo, after mushroom poisoning caused by an unknown species [9]. At the same time, press reports indicate that the cases of mushroom poisoning in Germany are on the rise since the beginning of the refugee crisis, e.g., five asylum seekers from Eastern Europe received medical treatment after eating *Amanita phalloides*. The same article mentions that in 2016 30 asylum seekers were hospitalized in one of Hanover’s hospitals after eating death cap mushrooms [10].

### Amanita phalloides

The most serious food poisonings are caused by lamellar mushrooms, and the most dangerous of that kind is *Amanita phalloides*, also known as the death cap; this species has been responsible for up to 90–95% of deaths caused by poisonous mushroom ingestion in Poland [11, 12]. In Europe, this kind of mushroom is often mistaken for edible mushrooms, such as *Russula virescens, Tricholoma equestre*, or *Macrolepiota procera* [13], but the problem is worldwide [14–16]. While *A. phalloides* mushrooms can emit an unpleasant odor of cat urine after drying, they are tasty and have a sweet scent when fresh and therefore may not raise any suspicion that they may be dangerous when consumed. Most importantly, thermal processing does not neutralize the toxins present [17].

Children are more likely to demonstrate a severe and often fatal course of poisoning [11, 18–20]. A typical example is the event that took place at the end of August 2021, when several Afghan citizens evacuated from Kabul and located in a refugee camp in Poland became poisoned with *A. phalloides*. Two boys, aged 5 and 6 years, died despite treatment.

Our article presents a case of poisoning with *Amanita phalloides* in a 28-year-old man and the incorrect medical procedures of the medical emergency unit. It is important for healthcare professionals to be aware of the possibility of mushroom poisoning and its potentially fatal consequences.

### Symptoms and mechanism of toxicity

It is believed that a single fruiting body of *A. phalloides* weighing approximately 50 g may contain a lethal dose for an adult human (about 0.1 mg per kg body weight) [19]. The key toxic agent is α-amanitin, an RNA polymerase II inhibitor [15, 17]. Unfortunately, *A. phalloides* poisoning is characterized by a delayed onset of symptoms and, hence, an increased risk of complications.

The initial symptoms usually appear within the first 8–24 h of ingestion. The toxin has a great affinity for fast-dividing cells, such as hepatocytes, intestinal villi cells, renal tubular cells, and lymphocytes. The most common symptoms include nausea, vomiting, general weakness, severe abdominal pain, headaches, and watery cholera-like stools [17]. However, it should be noted that these symptoms are not pathognomonic and may also be associated with other disorders. Physical examination of the patient will reveal tenderness in response to palpation of the epigastrium, as well as hepatomegaly. This symptomatic stage may be followed by an apparent improvement, which may last even up to 2 days; however, the symptoms invariably recur and at this point the patient typically seeks medical help. The concentration of liver indicators (transaminases and bilirubin) typically increases 24 h after mushroom ingestion and liver failure is observed after 4 to 5 days [19].

### Diagnosis

The diagnosis is based on an in-depth interview of the patient, with particular consideration paid to the time of consumption and the amount of mushrooms ingested, and a urine test to determine the presence of amanitin. Amatoxins are rapidly absorbed in the digestive system. They may be present in the blood for up to 36 h and in the urine for a couple of days. If possible, the leftovers of the mushroom meal consumed, or the vomitus of a sick person should be preserved for mycological examination. However, such analyses may be difficult to perform, especially when only vomitus is available, because mushrooms will be in a changed form. Therefore, genetic testing-based methods are becoming increasingly popular to identify the species [17]. In fatal cases, autopsies usually reveal signs of jaundice, cerebral oedema, subserosal petechiae, pulmonary congestion, liver steatosis, renal congestion with ecchymosis, and hemorrhages in the cortical part.

## Case presentation

While on a morning mushroom picking expedition in the forest, the 28-year-old victim found three specimens that he believed to be parasol mushrooms (*Macrolepiota procera*). After returning from the forest at about 10 a.m., he consumed them fried in oil. In the late afternoon, he began to complain of nausea and a feeling of heaviness in the stomach. This aroused his suspicion that he could have eaten *Amanita phalloides*.

Around 7 p.m., with abdominal pain worsening, the man called a medical rescue team, stating that he had consumed mushrooms similar to *Macrolepiota procera* found in the forest in the morning. On examination, the patient demonstrated abdominal pain at palpation in the gastric region and high blood pressure (160/100 mmHg). The head of the rescue team established a diagnosis of abdominal pain and primary hypertension. The patient received ranitidine 100 ml i.v. (intravenously) and captopril 12.5 mg p.o. (orally). The decision was made to leave the patient at home with the recommendation to consult a family doctor due to increased blood pressure.

Shortly after leaving the medical rescue team, the patient experienced vomiting, which persisted until the next day, when new symptoms appeared: diarrhea and subpyretic state. The patient contacted the ambulance medical dispatcher again and reported the new symptoms. The dispatcher recommended hydration and refused to dispatch a medical emergency team.

The condition did not improve and the next day the patient called the ambulance again. He informed the same medical dispatcher that he had not been able to eat and had experienced vomiting and diarrhea for 3 days. The dispatcher instructed the victim to drink a lot of water again and suggested that he should travel to the hospital by his own means of transport, claiming that there was no free ambulance.

On the same day, around noon, the victim’s mother called the medical rescue team, reporting vomiting as a reason. At that time, the ambulance was sent to the patient. The head of the rescue team was the same paramedic who attended during the first visit. The interview showed that the patient had vomiting and diarrhea. This time, after undergoing medical rescue operations consisting of treatment with metoclopramide 10 mg i.v, isotonic multielectrolyte physiological fluid 0.5 l i.v and 0.9% NaCl 500 ml i.v, the patient, who was already in a life-threatening health condition, was transported to the hospital. Further tests found that he had signs of liver failure, possibly due to poisoning with *Amanita phalloides,* and he was referred for further diagnosis and treatment to a higher reference center after administration of 1200 mg of acetylcysteine. Due to life-threatening liver failure, he received a liver transplant from a deceased donor. After long-term treatment and rehabilitation, the patient was finally discharged home in a condition described as good.

## Discussion and conclusions

Mushroom poisoning is a serious food poisoning, but, compared to other toxic substances, it is at the bottom of statistical categories that measure the frequency of poisoning. It should also be noted that not all mushroom poisonings end in the death of the poisoned person, although the decisive factor influencing this is the speed of recognizing the harmful species of fungus and the fastest possible implementation of adequate therapy.

The collection of wild mushrooms is an integral part of the centuries-old tradition of Poland, which is shared with other Slavic nations and had been named ‘mycophylic’ by Russian researchers R. Gordon Wasson and Valentina Pavlovna Wasson [21]. This term refers to positive portrayal of mushrooms in culture and treating mushroom picking as part of national recreation. This tradition is in opposition to ‘mycophobic’ countries, such as Great Britain, Norway, Finland, Belgium, Denmark, or the USA, where the most popular type of mushrooms are cultivated champignons, although cases of mushroom poisoning are reported there too [22]. According to statistics the incidence of mushroom poisoning is higher in mycophylic countries, such as Poland [18], Russia, and China [23]. By contrast, deaths induced by mushrooms are rarely reported in the United States [15, 24], Canada [25], and Australia [26]. Nevertheless, the number of poisonings in mycophilic countries is lower than it might seem, because knowledge about mushrooms is greater in such countries, which means that although mushrooms are collected in large numbers, poisonings are not so common. However, the division presented above is currently changing due to the inflow of refugees, which forms a new challenge not only to medical care units, but also to services trained in providing proper information about the toxicity of local mushrooms and other flora species, which possibly may resemble edible species from refugee countries. In any case, such issues cannot be solely attributed to refugees, but have a wider dimension. Due to economic challenges brought about by the pandemic of COVID-19 and the ensuing and worsening social problems, people may try to consume unusual products.

Although not every case of *Amanita phalloides* poisoning ends in death or liver transplantation, as can be seen in a case of group poisoning in the Netherlands [22], it is crucial to quickly implement the correct medical procedure on suspicion of fungal poisoning: transport the poisoned person to hospital, perform toxicological tests and institute appropriate treatment. While the precise choice of treatment depends on the time of admission to the hospital, treatment options include gastric lavage, large doses of activated carbon, moderately enhanced diuresis, silibin, penicillin G, N-acetylcysteine. In the cases of fulminant hepatitis, the only treatment available is liver transplantation [20]. Albumin dialysis in the MARS (Molecular Adsorbents Recirculating System) may also be used in therapy [13, 27].

Research continues to identify more effective treatments to assist in patient recovery, including, among others, a promising compound – polymyxin B; the results of its in silico and in vivo animal tests are presented in a study from 2015. The authors conclude that the ideal therapeutic approach against *Amanita phalloides* intoxication would be to displace the amatoxins and/or complete their binding to RNA polymerase II without impairing its normal transcription activity. The results were encouraging and the authors report that further human studies are planned [28].

In conclusion, in cases of fungal poisoning, it is crucial to quickly implement the correct procedure, as this can prevent the need for liver transplant or even death. This is a particularly important consideration for the first medical professionals to contact the patient, especially in cases where the patient reports mushrooms consumption and presents alarming symptoms of the gastrointestinal tract. Such situations cannot be underestimated and ignored.

## Data Availability

Not applicable.

## Abbreviations

i.v.: Intravenosa (Latin), intravenously
p.o.: Per os (Latin), orally
RNA: Ribonucleic acid
